# The Role of AGG Interruptions in the Transcription of *FMR1* Premutation Alleles

**DOI:** 10.1371/journal.pone.0021728

**Published:** 2011-07-19

**Authors:** Carolyn M. Yrigollen, Federica Tassone, Blythe Durbin-Johnson, Flora Tassone

**Affiliations:** 1 Department of Biochemistry and Molecular Medicine, School of Medicine, University of California Davis, Davis, California, United States of America; 2 Department of Public Health Sciences School of Medicine, University of California Davis, Davis, California, United States of America; 3 M.I.N.D. Institute, University of California Davis Medical Center, Davis, California, United States of America; Peninsula College of Medicine and Dentistry, University of Exeter, United Kingdom

## Abstract

Fragile X associated disorders are caused by a premutation allele in the fragile X mental retardation 1 gene (*FMR1*) and are hypothesized to result from the toxic effect of elevated levels of expanded *FMR1* transcripts. Increased levels of *FMR1* mRNA have indeed been reported in premutation carriers; however the mechanism by which expanded alleles lead to elevated levels of *FMR1* mRNA in premutation carriers is unknown. Within the CGG repeat tract AGG interruptions are found, generally 1–3 present in normal/intermediate alleles (6–54 CGG repeats) and usually 0–1 in premutation alleles (55–200 CGG repeats). They are present at specific locations, generally occurring after 9 or 10 uninterrupted CGG repeats [(CGG)_9_AGG(CGG)_9_AGG(CGG)_n_ ]. We evaluated both the number of AGG interruptions and the resulting length of the uninterrupted 3′ CGG repeat pure tract in premutation alleles derived from two large cohorts of male and female carriers to determine whether the presence of AGG interruptions or the length of a pure stretch of CGG repeats influence the levels of *FMR1* mRNA in blood. Our findings indicate that neither the number of AGG interruptions, nor their position along the CGG tract have a significant affect on mRNA levels in premutation carriers. We also, as expected based on previous findings, observed a highly significant correlation between CGG repeat number (as both total length and length of pure CGG stretch) and *FMR1* mRNA expression levels, in both males and females. Importantly, we did not observe any significant difference in *FMR1* mRNA levels in premutation carriers based on age.

## Introduction

Expansion of the CGG trinucleotide repeat in the 5′ untranslated (UTR) region of the *FMR1* gene is causative for a number of disorders including fragile X syndrome (FXS), fragile X associated tremor ataxia syndrome (FXTAS) and *FMR1* related primary ovarian insufficiency (FXPOI) [1]. A CGG expansion in the *FMR1* gene has also been associated with an increased incidence of several other clinical involvements ranging from anxiety to autism spectrum disorder (ASD) [2]. The length of the repeat falls into several categories, defined by The American College of Medical Genetics [3], which are useful as molecular indicators and based on predicting the risk of expansion in the next generations. Individuals with an allele within the normal range (5–44 CGG repeats) are considered unaffected, while full mutation alleles (>200 CGG repeats) result in transcriptional silencing of the *FMR1* gene, absence of fragile X mental retardation 1 protein (FMRP) and fragile X syndrome. Intermediate (45–54 CGG repeats) and premutation alleles (55–200 CGG repeats) were originally characterized as having an increased risk of expansion in future generations, but having no phenotypic effect on the carrier of these alleles. Within the last decade, biological effects have been identified and studied [4], [5], [6], but more needs to be learned about the mechanism that leads to the clinical involvement in these transitional ranges.

Increased levels of *FMR1* mRNA correlating with the length of the repeat tract, have been reported in both male and female carriers of a premutation allele [7], [8], [9]. Although the elevated levels result from an increase in transcriptional activity [10] a concomitant decreased protein expression, due to a deficit in translational efficiency especially in longer expanded alleles, was also reported [7], [8], [9], [11], [12]. Recently, increased FMRP expression levels have been reported in premutation carriers with an allele within the 80–89 CGG repeats range compared to those in the lower and in the higher premutation range [12].

Within the CGG repeat tract are interspersions of AGG segments that occur in normal, intermediate, and premutation range alleles and commonly occur after 9 or 10 uninterrupted CGG repeats [(CGG)_9_AGG(CGG)_9_AGG(CGG)_n_ ]. These AGG interruptions can be stably inherited, such that the number of AGG interruptions and their position is likely to correspond in parent and offspring; however like the length of CGG repeat tracts in *FMR1*, there is a level of instability, such as the loss of an interruption during transmission [13].

Moreover, AGG interruptions have been predicted to increase the stability of the repeat tract during maternal transmission by decreasing the size of pure CGG repeats in the tract [14], [15]. AGG interruptions have been shown to affect both DNA and RNA structure at the *FMR1* locus. The DNA at a pure CGG repeat tract of *FMR1* has been shown to adopt a non-ß conformation, which is increasingly unstable in the presence of AGG interruptions and leads to the formation of bulges and larger loops [16]. The CGG repeat region is known to form secondary structures in DNA, such as hairpins and tetraplexes, and in RNA has also been shown to form hairpins, though tetraplexes were not observed [16].

In recent studies, the presence of AGG interruptions within the *FMR1* trinucleotide repeat has been investigated to determine if transcription or translation is affected [10], [12], [17]. Tassone et al. [10] looked at the effect of AGG interruptions on *FMR1* transcription levels and reported a lack of association between *FMR1* mRNA levels and the number of AGG interruptions in 147 subjects including 76 subjects with normal range alleles, 10 subjects with an intermediate allele and 59 subjects with a premutation allele. More recently, Peprah et al. [12] looked at the transcriptional and translational activity of *FMR1* and AGG interruptions in 74 premutation carriers and found no correlations, confirming previous findings from an *in vitro* system [17].

Here, we describe our results from an in-depth investigation of the influence of the number of AGG interruptions, their location within the CGG tract and the length of the 3′ CGG repeat pure tract on the level of *FMR1* mRNA in a cohort of 770 premutation carriers, including 288 males and 482 females. This study adds to the body of knowledge with an increased sample size for male participants and confirms the same findings in a cohort of female participants, which have not been previously studied. Additionally, using a new triple primer PCR, we have been able to precisely map AGG interruptions along the CGG repeat tract and to correlate their location with the *FMR1* mRNA transcriptional level. This new approach allowed us to analyze both the total length of the repeat region and the length of uninterrupted CGG repeats (a 3′ CGG repeat pure stretch). Previous analysis of the role AGG interruptions have on transcription may have been unable to detect a significant correlation because the analysis was based on the presence or the absence of AGG interruptions, but not on the number of uninterrupted CGG repeats.

## Results

DNA samples from 770 unrelated individuals comprising 288 males and 482 females were selected for a detailed molecular analysis including CGG repeat number, length of pure CGG stretch, activation ratio in females, AGG number and distribution, and *FMR1* mRNA expression levels. Males and females with the premutation were selected from fragile X pedigrees because they were known to carry the premutation (and confirmed by *FMR1* DNA testing). None were selected on the basis of clinical symptoms.

Of the 770 samples, 265 male and 435 female samples had measured mRNA transcript levels and 231 male and 397 female samples had information about the age of the participant at the time the DNA and RNA samples were collected in males and females respectively.

The mean age in male subjects at the time of sample collection was 45.8 years (±26.3), and the mean age of female subjects was 42.6 years (±16.8). Most of the female premutation carriers were identified consequent of having a child with FXS and male premutation carriers were part of projects focused on fragile X associated disorders (distributions included in Figure S1a,b).

**Figure 1:**
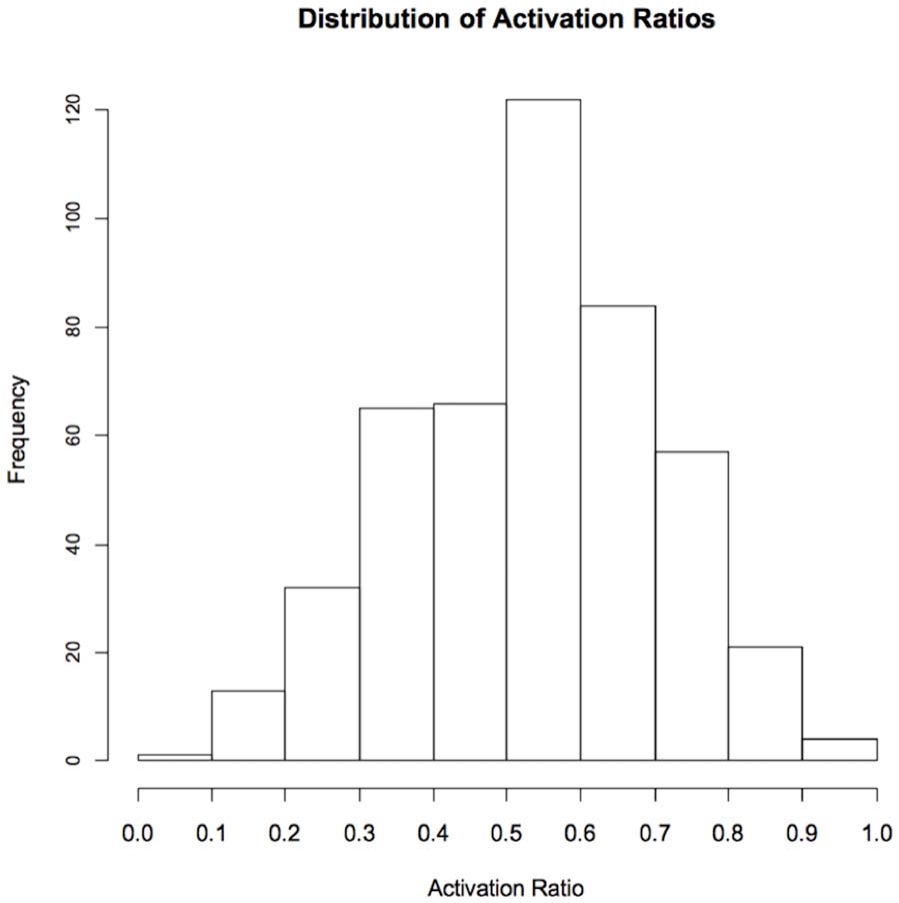
Distribution of activation ratio. Activation ratio was determined by Southern blot analysis. The distribution approximates a normal distribution with a mean of 0.54.

The mean *FMR1* mRNA level in males (3.1±1.2) was significantly different from the mean in the females (2.4±0.8) (t test P<0.001) due to the contribution of the normal allele on the other X chromosome (Table 1; distributions are presented in Supplementary Material (Figure S2a,b). CGG repeat allele sizes were comparable between males (mean 93.1±25.8) and females (89.2±20.7) (t test P = 0.035) (Figure S3a,b). The distribution of Activation ratio measured in 465 females had a mean of 0.54 and followed a normal distribution (Figure 1). Detailed molecular measures are presented in Table 1.

**Table 1:** A comparison of age, CGG repeat size, and *FMR1* mRNA levels in male and female premutation carriers.

	Male	Female
Samples (N)	288	482
Mean age	48.8	42.6
Age range	1–89	1–95
Mean Total CGG length (standard deviation)	93.1 (25.8)	89.2 (20.7)
Total CGG size range	55–220	55–190
Mean 3′ pure CGG stretch	84.9	82.6
3′ Pure CGG range	34–220	35–190
Number of AGG interruptions range	0–2	0–3
Mean *FMR1* mRNA levels	3.1	2.4
*FMR1* mRNA levels	1.1–9.0	0.8–6.4
Mean activation ratio		0.54
Activation ratio range		0.08–0.96

Of the 288 male samples, 23 had missing data on *FMR1* mRNA levels, 6 were missing the number of AGG interruptions and length of the longest pure CGG stretch, and 57 samples were missing participant age at time of sample collection. Missing observations were excluded from analyses as needed. The *FMR1* mRNA level from one additional sample was excluded from analyses due to an excessively large standard error of replicate measurements.

Of the 482 female subjects with the *FMR1* premutation allele included in the study, 47 samples had no data on *FMR1* mRNA levels, 17 samples did not have the activation ratio, and 85 samples were missing data on the participant age at time of sample collection. Missing observations were excluded from analyses as needed. The *FMR1* mRNA levels in females are determined by two alleles. In this study one allele was within the premutation range and the other was within the normal range (with the exception of 2 subjects, one with 2 premutation alleles and one with an intermediate and a premutation allele). Adjusted *FMR1* mRNA levels (estimates of mRNA_hp_, the contribution to the observed mRNA level from expanded alleles) were derived from the observed *FMR1* mRNA level and the activation ratios using the method described in Tassone et al. [9].

### FMR1 mRNA levels correlate with both total CGG repeat length and the length of 3′ pure CGG stretch in male and female carriers

**Figure 2:**
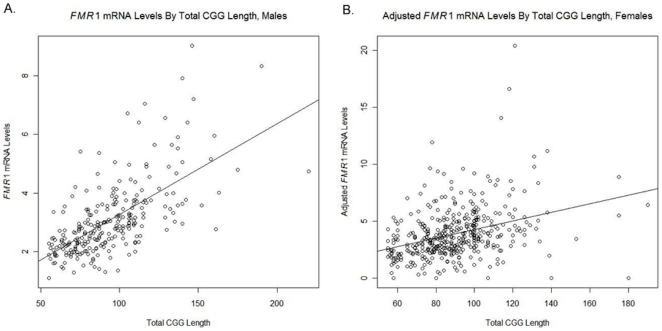
Scatterplot of *FMR1* mRNA levels by total CGG length. A linear regression model with model fit using CGG length as a continuous variable a) in premutation males and b) in premutation females. *FMR1* mRNA levels show strong (P<0.001) correlation to total CGG length.

In order to assess the relationship between *FMR1* mRNA levels and total CGG length, a linear regression model, with *FMR1* mRNA level as the response variable and total CGG length (as a continuous variable) as the predictor, was used. This and subsequent analysis was conducted separately on data from males and females. Based on the linear regression model fit using total CGG length as a continuous variable, *FMR1* mRNA levels appear to be strongly related to total CGG length (P<0.001) in both males (Figure 2a) and females (Figure 2b) confirming previous reports [7], [8], [9], [10], [12].

**Figure 3:**
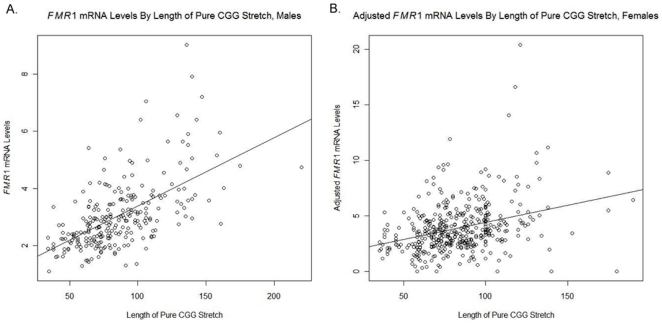
Scatterplot of *FMR1* mRNA levels by length of CGG pure stretch. A linear regression model with model fit using CGG pure stretch as a continuous variable a) in premutation males and b) in premutation females. *FMR1* mRNA levels appear strongly related to pure CGG stretch (P<0.001).

In order to assess the relationship between *FMR1* mRNA levels and length of 3′ pure CGG stretch the same analyses were conducted. Using a linear regression model, with *FMR1* mRNA level as the response variable and length of pure CGG stretch (as a continuous variable) as the predictor, *FMR1* mRNA levels appear to be strongly related to the length of 3′ pure CGG stretch (P<0.001) in both males (Figure 3a) and females (Figure 3b). *FMR1* mRNA levels were adjusted for activation ratio. Results were confirmed using an ANOVA model, with *FMR1* mRNA level as the response variable and length of pure CGG stretch as the predictor.

### The number of AGG Interruptions correlate with total CGG Length

**Figure 4:**
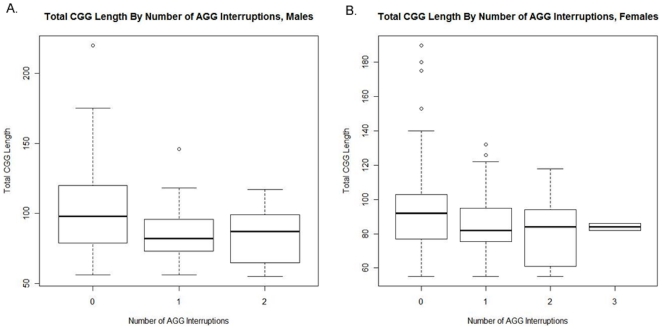
A box plot of total CGG length for each number of AGG interruptions. A) In premutation male data, significant difference in total CGG length of subjects with no AGG interruptions (130 subjects) and those with 1 or 2 interruptions (96 and 55 subjects respectively) was found (P<0.001 in both cases). No significance was found in total CGG length of subjects with 1 AGG interruption compared to 2 AGG interruptions (P = 0.935). B) Premutation female data showed significant difference in total CGG length of subjects with no AGG interruptions (259 subjects) and those with 1 or 2 interruptions (143 and 78 subjects respectively) (P<0.001) but no difference with remaining pairs.

The relationship between total CGG length and number of AGG interruptions was assessed using an ANOVA model, with total CGG length as the response variable and number of AGG interruptions as the predictor. The test for an AGG effect was highly significant (P<0.001) in both genders, rejecting the null hypothesis that mean total CGG length is equal in each AGG group. Tukey HSD post-hoc tests were conducted in order to test for pairwise differences between each group. In males, the total CGG length differed significantly between subjects with 0 interruptions and those with 1 or 2 interruptions (P<0.001 in both cases). However, the data did not suggest a difference in total CGG length between subjects with 1 AGG interruption and those with 2 AGG interruptions (P = 0.940). A box plot of total CGG length for each number of AGG interruptions is shown in Figure 4a. In females, the total CGG length differed significantly between subjects with 0 and 1 interruption (P = 0.001) and between those with 0 and 2 interruptions (p<0.001). However, the data did not suggest a difference in total CGG length between the remaining pairs of groups (note that this lack of significance should be interpreted cautiously for comparisons between patients with 3 interruptions and those with other numbers of interruptions due to the small sample size). A box plot of total CGG length for each number of AGG interruptions is shown in Figure 4b. Finally, no difference was observed in the interspersion pattern of AGG interruptions within the premutation alleles as already reported [13].

### FMR1 mRNA Levels do not correlate with the number of AGG interruptions

**Figure 5:**
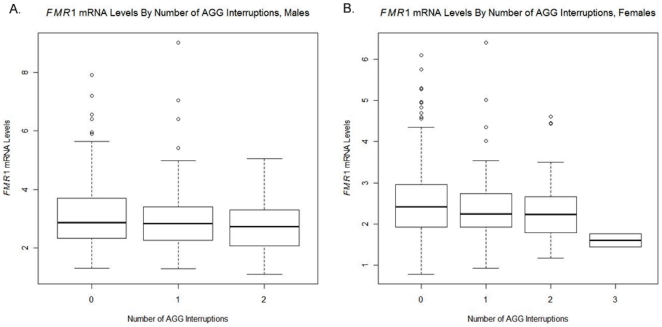
A box plot of *FMR1* mRNA levels for each number of AGG interruptions. No difference was found in *FMR1* mRNA levels between any of the AGG interruption groups a) in males (P = 0.060) or b) females (P = 0.066).

The relationship between *FMR1* mRNA levels and number of AGG interruptions was assessed using an ANOVA model, with *FMR1* mRNA level as the response variable and number of AGG interruptions as the predictor. The global test for an AGG effect did not suggest differences in *FMR1* mRNA levels between any of the AGG groups (P = 0.060 in males and P = 0.066 in females) (Figure 5a,b). These findings confirmed previous reports [10].

**Figure 6:**
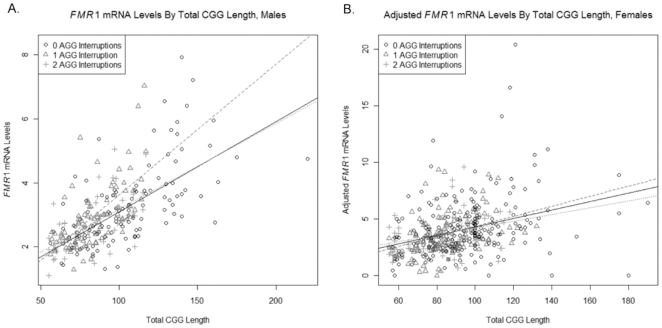
Scatterplot of *FMR1* mRNA levels by total CGG length. A linear regression model showed no significant difference in the slopes for 0, 1, or 2 AGG interruptions a) in males (P>0.046) or b) females (P>0.448). See also Table 2.

Given that the total CGG length, which is highly predictive of *FMR1* mRNA levels, differs significantly between subjects with different number of AGG interruptions, further analyses were conducted in which a separate linear regression of *FMR1* mRNA levels on total CGG length was fitted for each number of AGG interruptions. Differences in the intercepts of these lines would suggest differences in *FMR1* mRNA levels between subjects with different numbers of AGG interruptions but comparable total CGG length, whereas differences in the slopes of these lines would suggest that the impact of total CGG length on *FMR1* mRNA levels differs based on the number of AGG interruptions. In male subjects, the slope of the regression line fitted to data from subjects with 0 AGG interruptions showed a marginally significant difference from the slope of the regression line fitted to data from subjects with 1 interruption (P = 0.046); no other significant differences in slopes or intercepts of regression lines were seen for male or female subjects. (Analyses on data from female subjects used adjusted mRNA levels and combined 2 and 3 AGG interruptions into a single category.) When controlling for total CGG length, the number of AGG interruptions does not appear to have a meaningful impact on *FMR1* mRNA levels for both genders. (Figure 6a,b; and Table 2)

**Table 2:** Comparison of linear regression fits for *FMR1* mRNA levels by total CGG length.

Comparison	P-Value, Males	P-Value, Females
0 AGG vs. 1 AGG, Slope	0.046	0.637
0 AGG vs. 2 AGG, Slope	0.840	0.617
1 AGG vs. 2 AGG, Slope	0.101	0.449
0 AGG vs. 1 AGG, Intercept	0.191	0.626
0 AGG vs. 2 AGG, Intercept	0.708	0.642
1 AGG vs. 2 AGG, Intercept	0.185	0.448

### FMR1 mRNA levels are not affected by age

**Figure 7:**
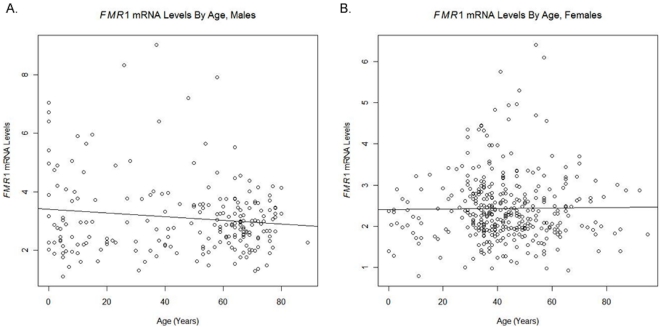
Scatterplot of *FMR1* mRNA levels by age. A linear regression model showed no significant relationship between *FMR1* mRNA levels and age, controlling for CGG length, in a) males (P = 0.093) or b) females (P = 0.297).

The relationship between *FMR1* mRNA and age, controlling for CGG, was assessed using a linear regression model, with *FMR1* mRNA level as the response variable and total CGG length (continuous) and age as predictors. Although the total CGG length remains strongly predictive of *FMR1* mRNA levels when age is included in the model (P<0.001) for both genders, our data do not suggest a statistically significant relationship between *FMR1* mRNA levels and age in either males or females when CGG is controlled (P = 0.100 in males and P = 0.297 in females). The scatter plots of *FMR1* mRNA levels by age are shown in Figure 7a,b.

## Discussion

Premutation alleles (55–200 CGG repeats) are relatively common in the general population with a prevalence of approximately 1 in 113–259 females and 1 in 251–813 males [13], [18], [19], [20], [21]. Although individuals with the premutation were previously thought to be clinically unaffected, it has become clear in the past decade that premutation carriers present with a number of clinical involvement including the fragile X-associated primary ovarian insufficiency (FXPOI) in approximately 20% of the females [22], [23], [24] and the fragile X-associated tremor ataxia syndrome (FXTAS) [25], [26], [27], [28] which occurs in approximately 1/3 of the male carriers and less frequently in female carriers. In addition, a variety of medical and developmental problems including ADHD and autism spectrum disorders (ASD) [29], [30] and psychiatric and emotional problems [31], [32], [33], [34], most notably depression and anxiety, are also commonly seen in premutation carriers. Psychological problems have been demonstrated to be significantly associated with an increased level of the *FMR1* mRNA [32] commonly observed in premutation carriers [7], [8], [9]. The increased levels of *FMR1* mRNA has led to the RNA gain- of- function model in which the *FMR1* mRNA itself is causative of the clinical problems observed in carriers, including FXTAS. Consistent with the mRNA toxicity model and the neurodegeneration associated with FXTAS, murine hippocampal neurons from CGG expanded neurons show reduced viability and display deficit in dendritic complexity including shorter dendritic length and fewer branches between 7 and 21 day compared to wild type age matched controls [35]. This provides evidence that disorders associated with the premutation genotype are the result of a global increase in *FMR1* mRNA levels that starts early in life. Our data showing no significant age-dependence on *FMR1* transcriptional levels is in agreement with these findings.

Although it has been demonstrated that the *FMR1* transcription rate, not the stability of the message, is increased in premutation alleles [9], [10] the mechanism by which this phenomena occurs has not been clarified yet. Thus, we have studied the potential role of AGG interruption and length of the 3′ CGG repeat pure stretch in a large cohort of male and female carriers of the *FMR1* premutation. Our findings indicate that *FMR1* transcription levels, which are elevated in premutation carriers, are not significantly altered by the presence or by the position of AGG interruptions. This comprehensive analysis compared the number of AGG interruptions within a premutation allele and *FMR1* mRNA levels and found significant difference between groups of AGG interruptions when the total length of the CGG tract was factored in. Genders were analyzed separately, adjustments were made for activation ratio in females, each gender comparison resulted in no correlation. These results are consistent with previous studies that compared *FMR1* mRNA levels by the number of AGG interruptions present in a given allele (Tassone et al., 2007). However, given that the total CGG length, which is highly predictive of *FMR1* mRNA levels, differs significantly between subjects with different number of AGG interruptions, further analyses were conducted in which a separate linear regression of *FMR1* mRNA levels on total CGG length was fitted for each number of AGG interruptions. When controlling for total CGG length, the number of AGG interruptions does not appear to have a meaningful impact on *FMR1* mRNA levels.

Finally, we have analyzed the correlation of *FMR1* mRNA levels and age in premutation carriers. This analysis was performed on both genders separately, and results were consistent between genders, as no significant association was made between age and mRNA level. Thus, the increase levels of *FMR1* mRNA in premutation carriers do not appear to change with time (either increasing or decreasing). However, *FMR1* transcript levels were determined in total RNA isolated from peripheral blood leukocytes. Thus, it is possible that the levels may change with time in other tissues (i.e. brain).

In conclusion, we have demonstrated that the elevated *FMR1* mRNA levels observed in premutation carriers, which are strongly correlated to the CGG repeat number, are not affected by the presence of the AGG interruption. Importantly we have shown that the *FMR1* mRNA levels in premutation carriers are not influenced by the age of the subject. This is of relevance as elevated levels of *FMR1* mRNA have been demonstrated to be causative or related not only to FXTAS, but also to the reported behavioral problems, cognitive impairment and seizure observed in early development in premutation carriers [27], [29], [30], [35], [36], [37] to autoimmune disorders in female premutation carriers [38], [39], [40] and perhaps to the primary ovarian insufficiency (FXPOI), observed in ∼20% female carriers of a premutation [23], [24], [41].

Thus, the elevated levels of *FMR1* mRNA, which appear to not change with time, are likely to be causative for both neurodevelopmental and neurodegenerative phenotypes in premutation carriers throughout their life.

## Materials and Methods

### Subjects

Individuals were recruited through the M.I.N.D. Institute Clinic and provided informed consent for participation. Individuals were selected from fragile X pedigrees based on molecular testing identifying a premutation size allele. None were selected on the basis of clinical symptoms.

Blood was collected in EDTA (for DNA isolation) and Tempus vacutainers (for total RNA isolation) under protocols approved by the Institutional Review Board. Samples were chosen for inclusion in this study based on the CGG repeat allele within the size range of a premutation. In total, 770 DNA samples, comprising 288 males and 482 females, were selected to be molecularly characterized (including CGG repeat number, length of 3′ pure CGG stretch, activation ratio, *FMR1* mRNA expression levels and presence of AGG interruptions).

### Molecular measures

#### CGG repeat size

Genomic DNA (gDNA) was isolated from 5 mL of peripheral blood leukocytes using Gentra Puregene Blood Kit (Qiagen). gDNA was used to determine the size of the CGG repeat allele, methylation status and the *FMR1* activation ratio in females by Southern Blot analysis and PCR amplification, using established diagnostic protocols [42].

#### *FMR1* mRNA levels

Total RNA was isolated from whole blood collected in Tempus vacutainers (Applied Biosystems). Reverse transcription followed by real time PCRs (qRT-PCR) to measure the *FMR1* transcript levels were performed on total RNA samples, as previously described [9].

The relative abundance of *FMR1* mRNA was assessed using the β-glucoronidase (*GUS*) mRNA as a reference gene. For each sample, RT reactions were run starting from 3 different total RNA concentrations (500–250 and 125 ng) and quantitative-PCR reactions were performed for both *FMR1* and *GUS* amplicons for a total of 12 reactions. Control reactions (*FMR1* and *GUS*) were run in parallel with total RNA isolated from a standard control lymphoblastoid used as a calibrator (RPM 7666; ATCC CCL114]). Details of the methodology and analysis are as described in [9].

#### AGG interruptions

**Figure 8:**
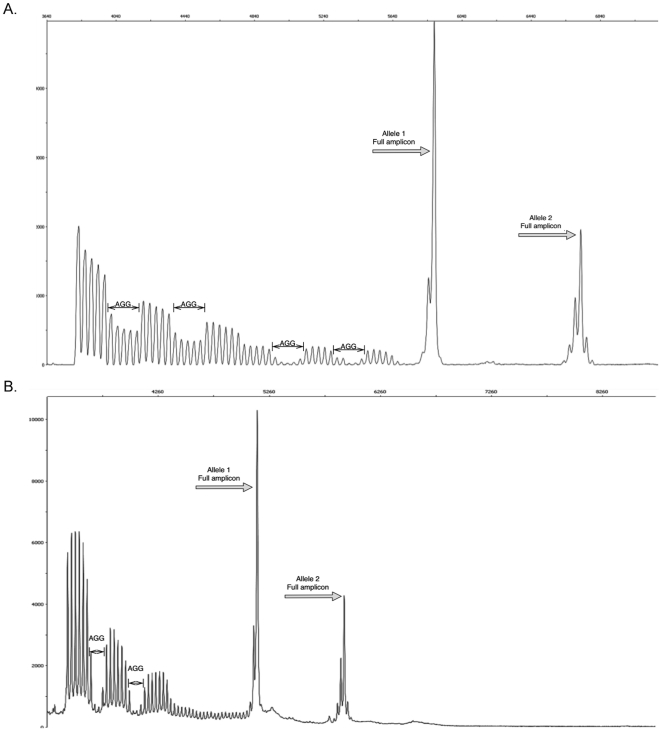
Electropherogram of FMR1 CGG linker PCR. a) PCR product from a premutation female with 1 normal allele (Allele 1) with 30 CGG repeats and 1 premutation allele (Allele 2) with 55 CGG repeats. Using a CCG chimeric primer, AGG interruptions can be identified as loss of signal intensity spanning 5 CGG peaks, followed by restoration of signal intensity. AGG interruptions occurring in the normal allele have a loss of approximately half the CGG peak intensity due to the presence of CGG peaks from the premutation allele. Allele 1: (CGG)_10_-AGG-(CGG)_9_-AGG-(CGG)_9;_ Allele 2: (CGG)_9_-AGG-(CGG)_9_-AGG-(CGG)_35_ . b) PCR product using a GGC chimeric primer and the same DNA sample as Fig. 8a. AGG interruptions result in loss of signal in 4 consecutive CGG peaks, followed by restoration in signal. Overlapping AGG interruptions results in peak intensity falling to baseline levels.

A PCR approach including a CGG repeat primer, as previously described [35], [42] was used to determine the number and the distribution of AGG interruptions within the *FMR1* gene. PCR was performed using 100 ng of gDNA following manufacturer's recommendations (Asuragen) and as described in Chen et al., [35]. The presence and the distribution of AGG interruptions was determined by the analysis of an electropherogram as illustrated in Figure 8a and in Figure S4. Briefly, a three primer assay is designed to amplify the 239 bp flanking region of the trinucleotide repeat (using c and f primers) [43], resulting in a PCR fragment corresponding to the CGG repeat size. The third primer, a CGG chimeric primer (CCG)_5_ which anneals to CGG tracts, amplifies intermediate fragments that are variable in length. The chimeric primer is unable to anneal to a CGG tract that is interrupted with an AGG, this allows the location of interruptions to be mapped by identification of a corresponding absence of PCR product.

gDNA was amplified in a 15 µl total reaction volume using ABI GeneAmp 9700 (Applied Biosystems) under the following conditions: 95°C for 5 minutes; followed by 10 cycles of 97°C for 35 seconds, 62°C for 35 seconds, 68°C for 4 minutes; 20 cycles of 97°C for 35 seconds, 62°C for 35 seconds, 68°C for 4 minutes and an additional 20 seconds per cycle; 72°C extension for 10 minutes and finally incubation at 4°C. Two microliters of PCR product was denatured with 2 µL of ROX 1000 size marker (Asuragen) in Hi-Di Formamide (Applied Biosystems) to a total volume of 15 µL for 2 minutes at 95°C followed by 3 minutes at 4°C. The single stranded amplified product was visualized by capillary electrophoresis using the ABI 3100 Genetic Analyzer (Applied Biosystems). Fragments were separated through a 36-cm capillary array using POP4 polymer (Applied Biosystems) following manufacturers recommendations with the following changes to the default run parameters: injection for 20 seconds at 1250 volts, and a run time of 50 minutes. AGG positions within the CGG element were determined from raw Genescan files imported into Peakscanner software (v1.0) (Applied Biosystems). FAM-6 labeled PCR product is displayed as an electropherogram. The full fragment visualized as a distinct band(s) amplified by the c and f primers is first identified and sized using the size standard marker (Asuragen). The CGG linker primer generates PCR fragments that are smaller than the sizing amplicons and are varying in size. They are identified on Peakscanner as peaks with lower signal intensity than sizing peaks of normal range alleles and are smaller fragment lengths than sizing peak that correspond to the same allele. The presence of an AGG interruption can be visualized on the electropherogram as a loss of signal intensity spanning 5 CGG peaks, followed by the restoration of signal intensity (Figure 8a,b). These 5 peaks correspond to the positions of the AGG interruption and 4 CGG segments up and downstream of the AGG interruption. Similarly, the first CGG peak on the electropherogram is the 5^th^ CGG repeat of the amplicon, the first 5 repeats being the earliest position on the DNA template for the CGG linker primer to anneal. To characterize the CGG tract the peaks and dips are ordered in reverse of their position on the electropherogram, given the directionality of the f and CGG linker primers. AGG interruptions are expected at the far end of the electropherogram, where signal intensity naturally decreases from longer PCR amplification being less thermodynamically favorable than shorter fragments.

A sample set including 47 DNAs underwent an additional PCR step to confirm the position of the AGG interruption using a (GGC)_4_ chimeric primer as described in Tassone et al., [42] (Figure 8b). In this case the linker anneals to the CGG tract on the reverse strand, and only requires 4 uninterrupted repeats. This primer amplifies in conjunction with the c primer, creating the same AGG interruption dips that were previously seen at the tail end of the electropherogram but now visualized at the beginning of the electropherogram with the highest signal intensity. It is to be noted that potential ambiguity of AGG position may exist in female samples in cases where the location of the AGG interruption could be attributed to the same position of both alleles (normal and premutation). There is a small risk for AGG interruptions of the premutation allele to be incorrectly interpreted as being present on the normal allele in females. The ambiguity of one AGG interruption in a location where two CGG repeat sequences overlap can typically be resolved in premutation carriers using the CGG linker because interruptions are expected to occur at the 3′ end of the CGG repeat. The CGG linker assay amplifies the 5′ end of the CGG repeat first which provides a length of space between the AGG interruptions on a premutation allele with the AGG interruptions of a normal allele. The cases where this may not be true are when the premutation allele is a low premutation and the normal is a high normal, also many AGG interruptions will decrease the space between premutation and normal AGG interruption ranges, and very rare AGG interruption patterns that are located more than 20 repeats from the 3′ end of the CGG repeat. In order to resolve these ambiguous female premutation carrier samples the typical CGG linker assay was used and the presence or absence of AGG interruptions was determined by digest with EciI as previously described [10].

## Supporting Information

Figure S1 **Distribution of age in participants.** The distribution of ages in a) males and b) females.
(TIFF)

Figure S2 **Distribution of** ***FMR1*** **mRNA levels.** Distributions of *FMR1* mRNA levels in a) males and b) females.
(TIFF)

Figure S3 **Distribution of CGG repeats in the** ***FMR1*** **gene.** Distributions of *FMR1* CGG repeats are similar in a) males and b) females.
(TIFF)

Figure S4 **Electropherogram of** ***FMR1*** **CGG linker PCR.** PCR product from a premutation female with 1 normal allele (Allele 1) with 2 AGG interruptions in 30 CGG repeats and 1 premutation allele (Allele 2) with 2 AGG interruptions in 87 CGG repeats. Determination of the number and location of the AGG interruptions within each allele was performed as described in the method section.
(TIFF)
