# Supplementary figures and images for: The Role of AGG Interruptions in the Transcription of FMR1 Premutation Alleles
Source: PLoS One. 2011 Jul 19;6(7):e21728. doi: 10.1371/journal.pone.0021728 (PMC3139575; doi:10.1371/journal.pone.0021728)

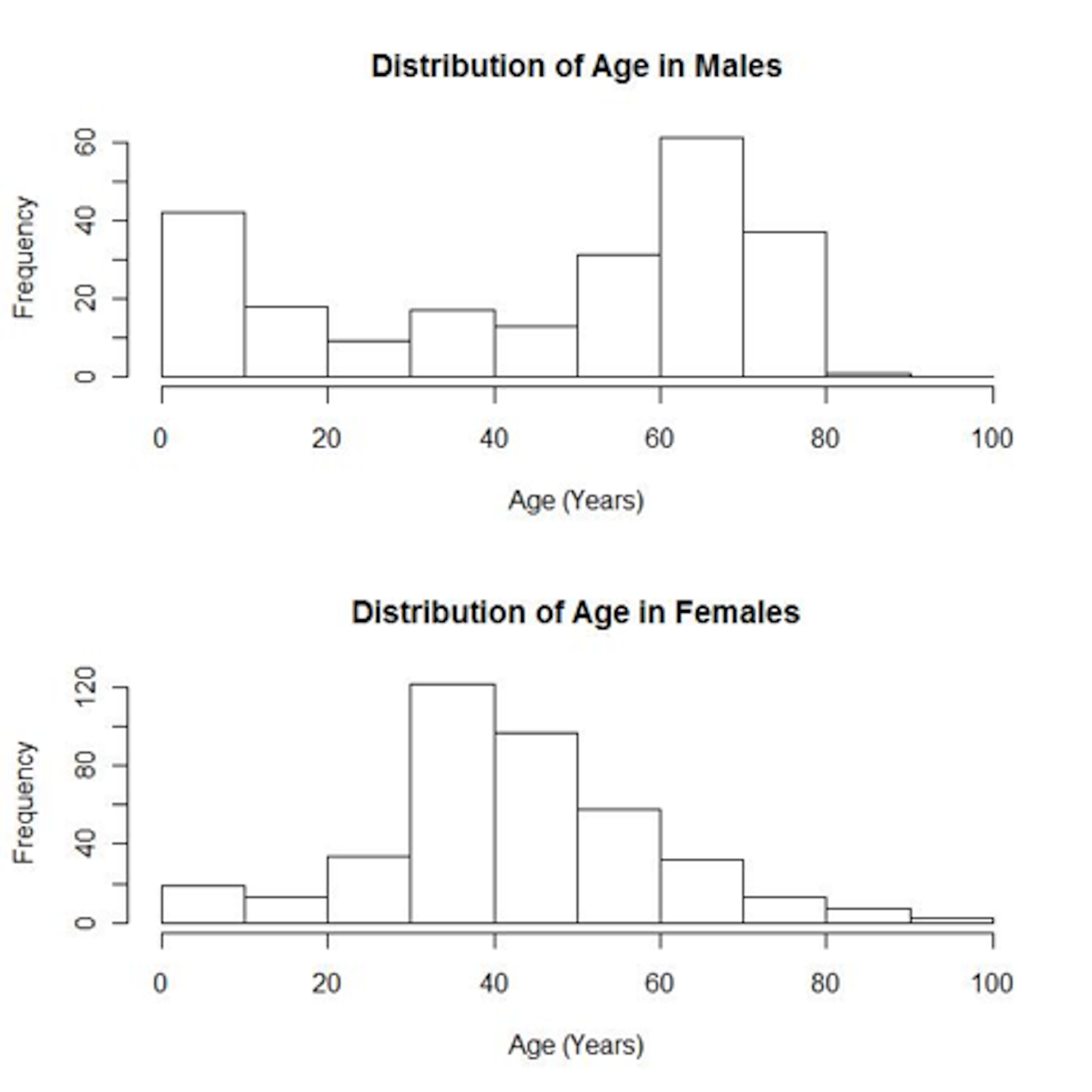

Supplement: Figure S1 — Distribution of age in participants. The distribution of ages in a) males and b) females. (TIFF) [file pone.0021728.s001.tiff]

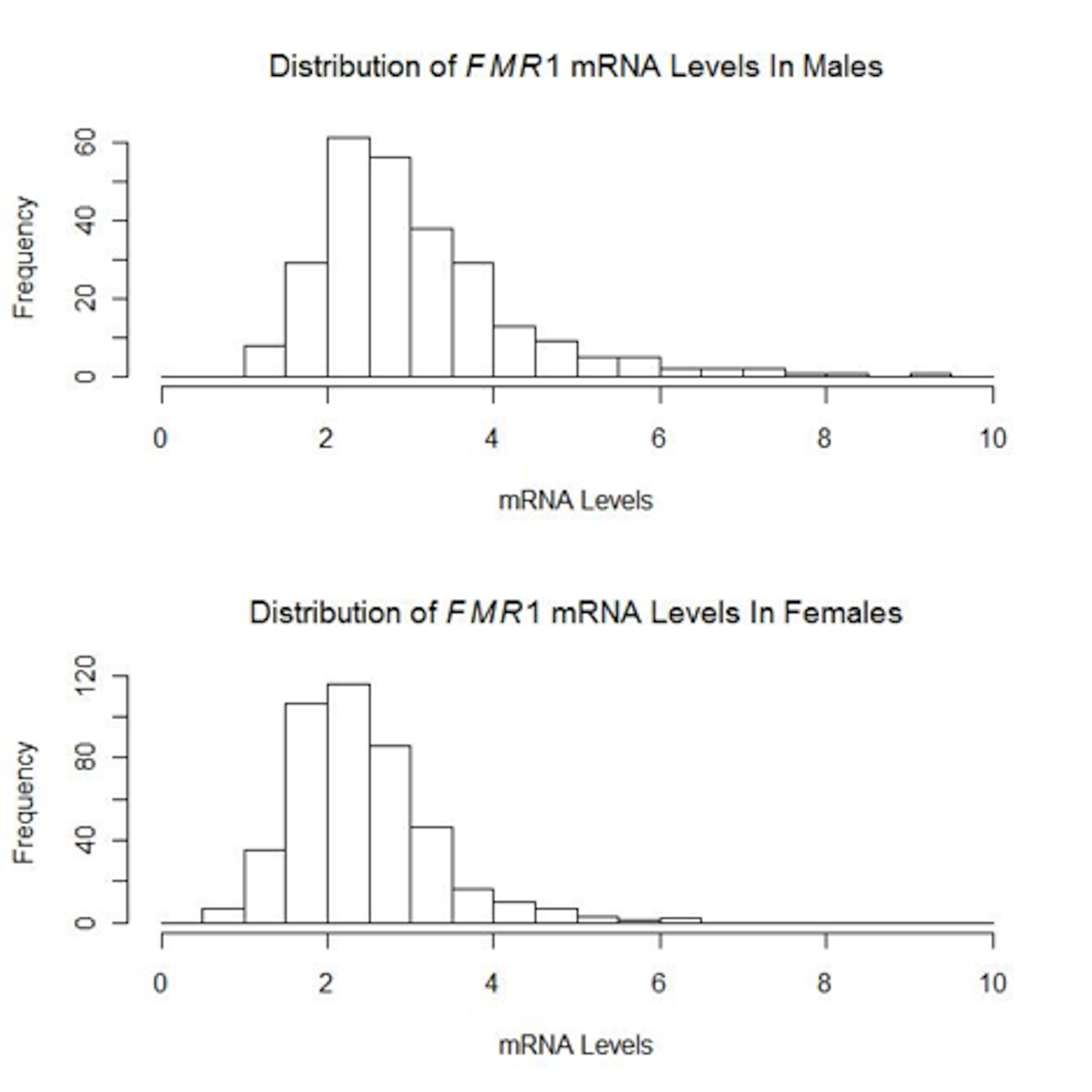

Supplement: Figure S2 — Distribution of FMR1 mRNA levels. Distributions of FMR1 mRNA levels in a) males and b) females. (TIFF) [file pone.0021728.s002.tiff]

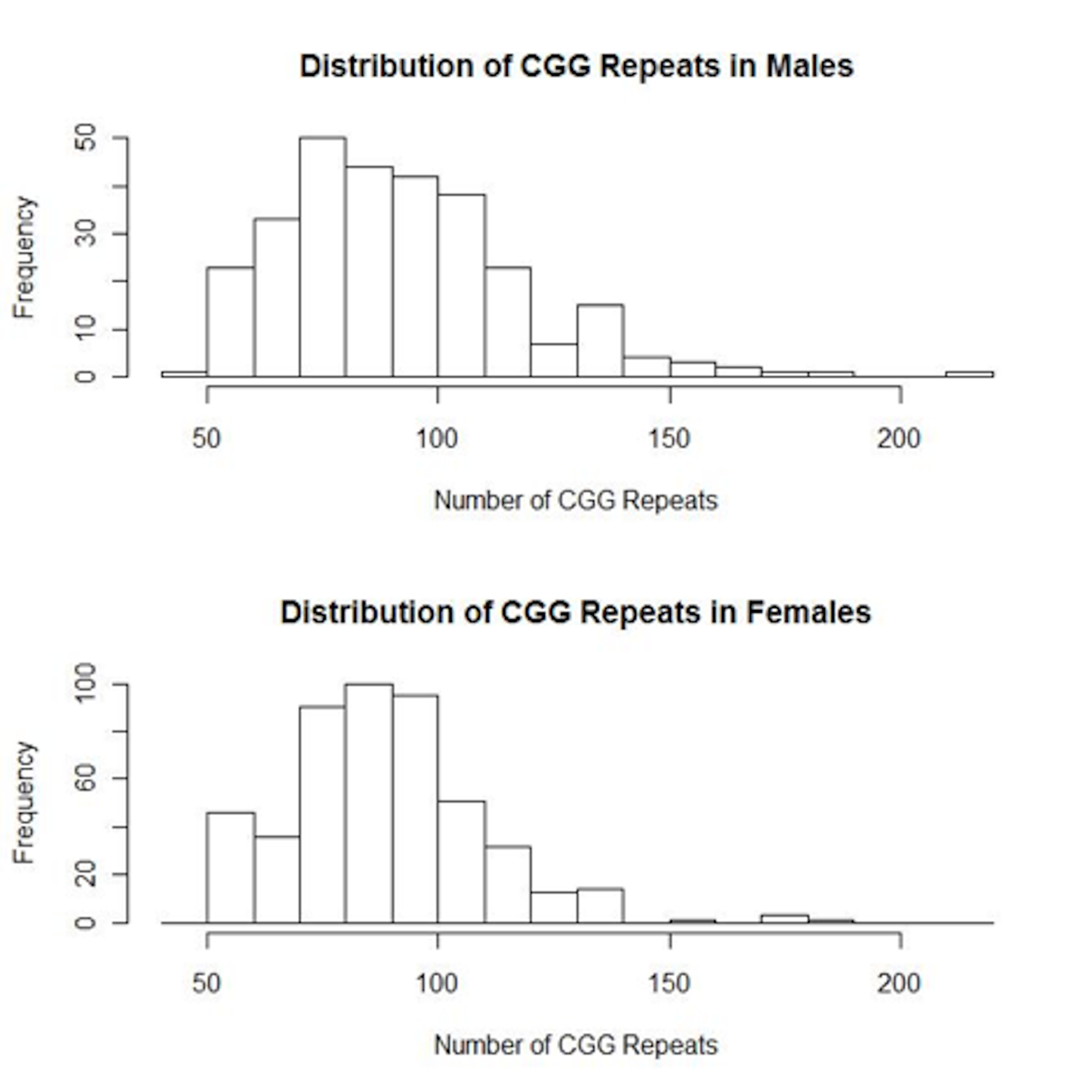

Supplement: Figure S3 — Distribution of CGG repeats in the FMR1 gene. Distributions of FMR1 CGG repeats are similar in a) males and b) females. (TIFF) [file pone.0021728.s003.tiff]

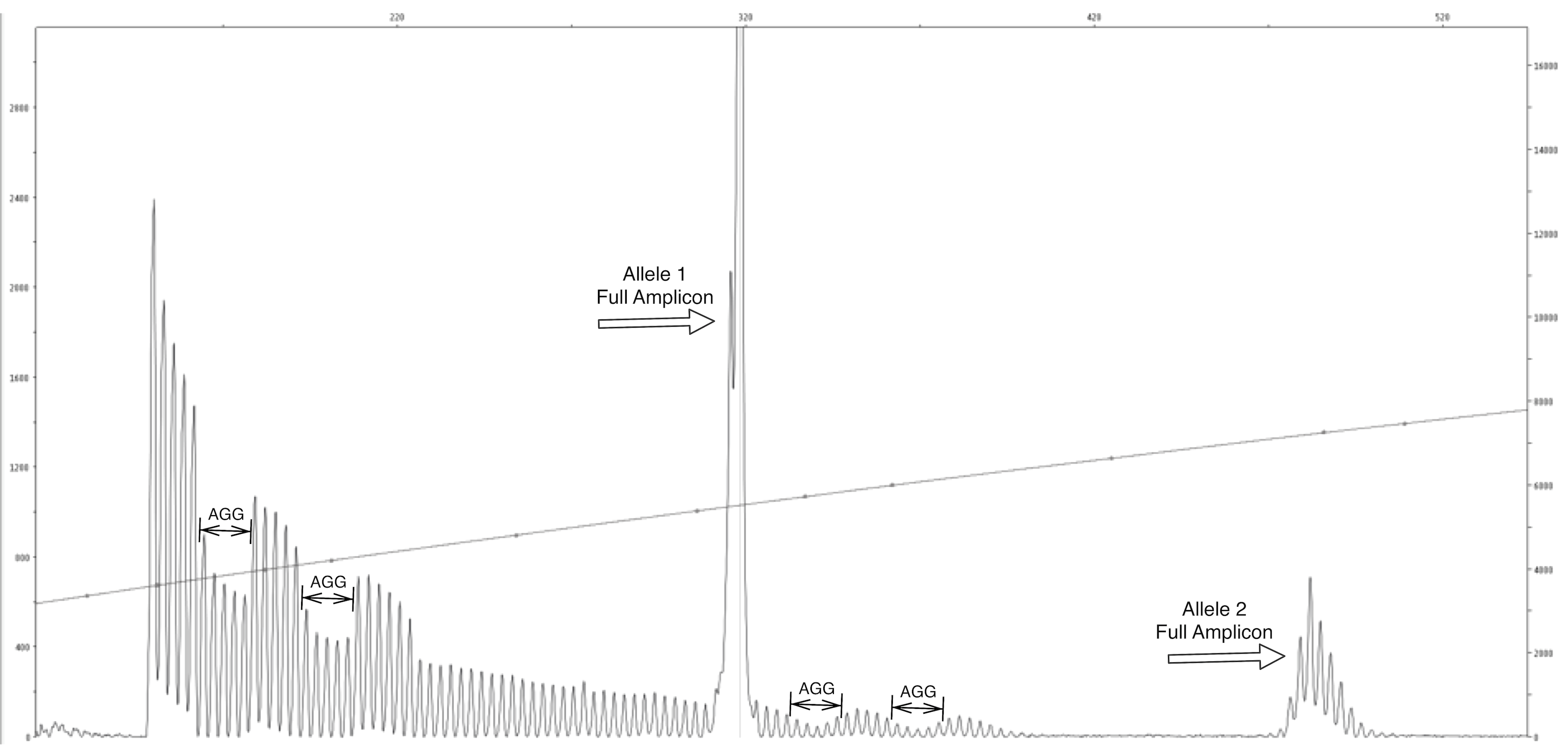

Supplement: Figure S4 — Electropherogram of FMR1 CGG linker PCR. PCR product from a premutation female with 1 normal allele (Allele 1) with 2 AGG interruptions in 30 CGG repeats and 1 premutation allele (Allele 2) with 2 AGG interruptions in 87 CGG repeats. Determination of the number and location of the AGG interruptions within each allele was performed as described in the method section. (TIFF) [file pone.0021728.s004.tiff]
